# Simultaneous Electrophysiology and Fiber Photometry in Freely Behaving Mice

**DOI:** 10.3389/fnins.2020.00148

**Published:** 2020-02-21

**Authors:** Amisha A. Patel, Niall McAlinden, Keith Mathieson, Shuzo Sakata

**Affiliations:** ^1^Strathclyde Institute of Pharmacy & Biomedical Sciences, University of Strathclyde, Glasgow, United Kingdom; ^2^Department of Physics, Institute of Photonics, SUPA, University of Strathclyde, Glasgow, United Kingdom

**Keywords:** brain state, REM sleep, GCaMP, acetylcholine, pontine waves, brainstem

## Abstract

*In vivo* electrophysiology is the gold standard technique used to investigate sub-second neural dynamics in freely behaving animals. However, monitoring cell-type-specific population activity is not a trivial task. Over the last decade, fiber photometry based on genetically encoded calcium indicators (GECIs) has been widely adopted as a versatile tool to monitor cell-type-specific population activity *in vivo*. However, this approach suffers from low temporal resolution. Here, we combine these two approaches to monitor both sub-second field potentials and cell-type-specific population activity in freely behaving mice. By developing an economical custom-made system and constructing a hybrid implant of an electrode and a fiber optic cannula, we simultaneously monitor artifact-free mesopontine field potentials and calcium transients in cholinergic neurons across the sleep-wake cycle. We find that mesopontine cholinergic activity co-occurs with sub-second pontine waves, called P-waves, during rapid eye movement sleep. Given the simplicity of our approach, simultaneous electrophysiological recording and cell-type-specific imaging provides a novel and valuable tool for interrogating state-dependent neural circuit dynamics *in vivo*.

## Introduction

Intracranial electrophysiological recordings monitor neuronal activity at various spatial scales, from single cells to populations across brain regions, with high temporal resolution (Buzsaki, 2004; Buzsaki et al., 2012; Jun et al., 2017). However, one of limitations in this approach is identifying the source of the neural signal: because neuronal activity is typically monitored extracellularly in freely behaving condition, the identification/isolation of recorded neurons is challenging (Einevoll et al., 2012; Harris et al., 2016).

Genetically encoded indicators offer complementary advantages over *in vivo* electrophysiological approaches (Lin and Schnitzer, 2016; Deo and Lavis, 2018; Wang et al., 2019). Over the last two decades, genetically encoded calcium indicators (GECIs) have been widely used to interrogate not only neuronal ensemble dynamics, but also activity of non-neuronal cells, such as astrocytes *in vivo* (Nakai et al., 2001; Chen et al., 2013; Stobart et al., 2018; Dana et al., 2019; Inoue et al., 2019; Stringer et al., 2019). For example, GECIs enable cell-type-specific targeting and long-term monitoring of neuronal activity *in vivo*. However, because of the intrinsic nature of calcium signals, the low temporal resolution of GECIs are not ideal for monitoring sub-second neural dynamics. It is also challenging to monitor individual neuronal activity in deep brain areas without causing significant tissue damage.

Here, we combine an electrophysiological approach with GECI-based fiber photometry to simultaneously monitor both field potentials and calcium transients in freely behaving mice. Fiber photometry is an imaging method used to monitor fluorescent signals via an implanted fiber optic cannula (Adelsberger et al., 2005; Lutcke et al., 2010; Kim et al., 2016; Sych et al., 2019). Although it is still invasive, the diameter of the cannula is thinner than an endoscope. Therefore, fiber photometry is well-suited to monitor neural population activity in deep tissue, such as the brainstem.

In the present study, we develop a versatile custom-made fiber photometry system with the capability to integrate *in vivo* electrophysiological recording in freely behaving mice. To validate our system, we focus on pontine waves (P-waves), which were reported in mice recently (Tsunematsu et al., 2020). Because mesopontine cholinergic neurons have been implicated in the induction of P-waves (Callaway et al., 1987; Datta, 1997), we monitor calcium transients from GCaMP6s-expressing mesopontine cholinergic neurons along with detecting P-waves electrophysiologically. We show that P-waves during REM sleep co-occurs with calcium transients in mesopontine cholinergic neurons. Thus, our system allows simultaneous electrophysiological recording and fiber photometry in freely behaving mice.

## Materials and Methods

### Recording System Configuration

**FIGURE 1:**
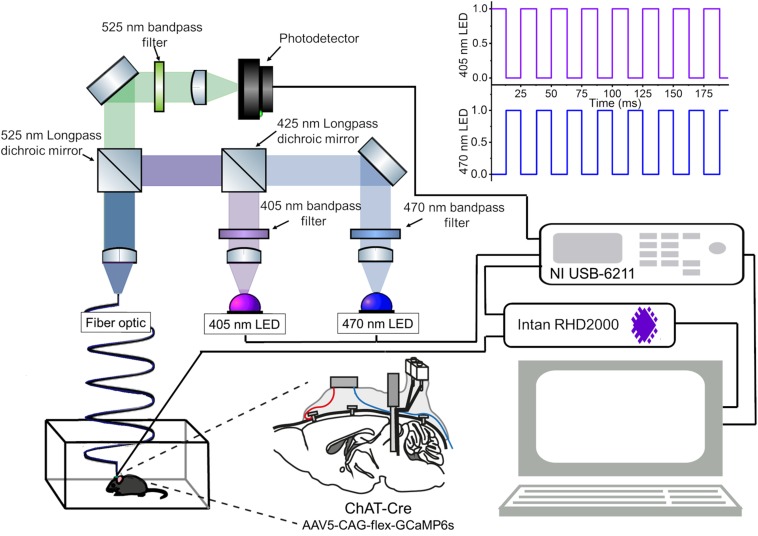
An integrated system of fiber photometry and electrophysiology. A schematic of the fiber photometry system with two LEDs. A detailed parts list of the optical setup is provided in Table 1. The data acquisition module (NI USB-6211) provides analog outputs to drive the LEDs with a 40 Hz alternated illumination pattern (*top right*), and obtains analog inputs from the photodetector for fluorescent signals and from the Intan system for electrophysiological signals (*bottom right*). Please note that the intensity of LED pulses is adjusted in every experiment. A construction manual of the optical setup is provided in Supplementary Material. Another schematic drawing of the mouse brain (*bottom center*) illustrates the implant configuration: cortical EEGs were monitored via bone screws implanted over the frontal cortex (red). EMGs were monitored via twisted wires (blue) inserted to the neck muscle. The implant is a hybrid bipolar electrode and optical fiber. In this study, we expressed GCaMP6s in mesopontine ChAT + neurons.

The recording system is shown in Figure 1 and a parts list for the fiber photometry system is summarized in Table 1. A detailed construction manual is provided in Supplementary Material. Essential codes for data acquisition and data processing are also available^1^.

**TABLE 1:** Parts list for the fiber photometry system.

Component	Supplier	Product code	Quantity
Photodetector	Newport (United Kingdom)	NewFocus 2151	1
470 nm LED		M470L3	1
405 nm LED		M405L3	1
LED driver		LEDD1B	2
LED power source		KSP101	2
LED holder		CP12	2
Dichroic mirror		DMLP425R	1
Dichroic mirror		MD498	1
Excitation filter		FB470-10	1
Excitation filter		FB405-10	1
Band-pass filter		MF525-39	1
Broadband dielectric mirror	Thorlabs (United Kingdom)	BB1-E02	2
Mirror mount		KCB1C/M	2
Aspheric lens		AL2520M-A	4
Cage plate for lens		CP08/M	4
Lens tubes		SM1L03-P5	1
Filter holder		C4W	2
Filter holder		B4C/M	2
Filter holder		FFM1	2
Fiber launch		KT110/M	1
Patch cable		M82L01	1
Optic fiber implant		CFM14L05-10	1
Mating sleeve		ADAF1-5	1
			

Briefly, the fiber photometry system consisted of two excitation channels. A 470 nm LED (M470L3, Thorlabs) was used to extract a Ca^2+^-dependent signal and a 405 nm LED (M405L3, Thorlabs) was used to obtain a Ca^2+^-independent isosbestic signal. Light from the LEDs was directed through excitation filters (FB470-10, FB405-10, Thorlabs) and a dichroic mirror to the fiber launch (DMLP425R and KT110/M, respectively). The fiber launch was connected to a multimode patch cable (M82L01, Thorlabs) which could be reversibly attached and detached to an implantable optic fiber on the mouse via a ceramic mating sleeve (CFM14L05 and ADAF1, respectively). Light emissions from GCaMP6s expressing neurons were then collected back through the optic fiber, and directed through a detection path, passing a dichroic mirror (MD498) to reach a photodetector (NewFocus 2151, Newport). A National Instruments DAQ (NI USB-6211) and custom-written LabVIEW software was used to control the LEDs and acquire fluorescence data at 1 kHz. LEDs were alternately turned on and off at 40 Hz in a square pulse pattern. Electrophysiology signals were recorded at 1 kHz using an interface board (RHD2000, Intan Technologies) and connected to the mouse via an amplifier (RHD2132 16-channel amplifier board, Intan Technologies).

### Implant Fabrication

**FIGURE 2:**
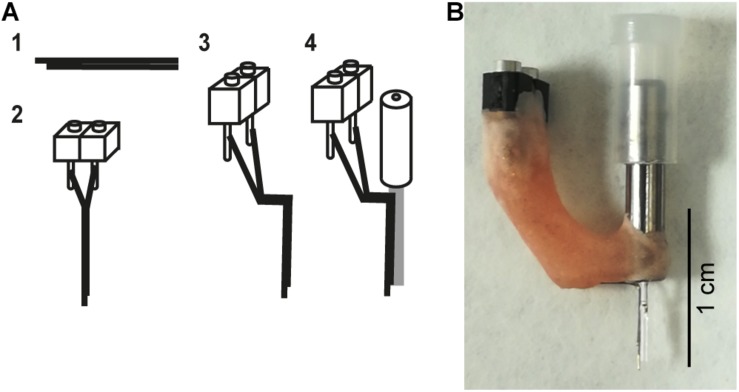
Hybrid implant. **(A)** The fabrication process for making hybrid implants. (1) Two wires are glued together to create a bipolar electrode. To differentiate local signals, the tip of two wires was separated by up to 1 mm. (2) The bipolar electrode is attached to a connector. (3) The bipolar electrode is bent. (4) The bent bipolar electrode is attached to a fiber optic cannula. **(B)** A photograph of an assembled implant.

Hybrid implants (also referred to as an optrode) consisted of a bipolar electrode (pair of electrodes) glued to the optic fiber and were fabricated though a multistep process. First two 0.1 mm diameter stainless steel wires (FE205850/2, Goodfellow) were cut to approximately 1.5 cm in length with fine scissors (14084-08, Fine Science Tools). Both wires were glued together (offset by approximately 0.5–1 mm at the tip) and the other end of the bundle was cut so that the tips of the wires were aligned (Figure 2A, step 1). Insulation was scraped off from the flush end of the bundle using a scalpel blade and connected to a 2-piece connector (SS-132-T-2-N, Samtec) using conductive epoxy (186-3593, RS Pro) (Figure 2A, step 2). The conductive epoxy was left to dry for 10 min and then secured with dental cement. Impedances were checked by connecting the bipolar electrodes to the Intan system (RHD2132 16-channel amplifier board and RHD2000, Intan Technologies) with a custom-made connector, and placing the tips of the bipolar electrodes in saline. Electrodes with impedances between 200 kΩ and 1 MΩ at 1 kHz were folded (Figure 2A, step 3): the vertical shaft closest to the electrode tips was approximately 5 mm long and the horizontal section 3–4 mm. The folded electrode was positioned alongside the optic fiber and fixed in place 500 μm below the tip of the optic fiber with superglue (473-455, RS Pro), taking care not to get glue on the tips of either the optic fiber or bipolar electrode (Figure 2A, step 4). Dental cement was then used to secure and stabilize the structure (Figure 2B). Impedances were checked again (the range was 276–452 kΩ) and optrodes were ready for implantation.

### Animals

All animal experiments were performed in accordance with the United Kingdom Animals (Scientific Procedures) Act of 1986 Home Office regulations and approved by the Home Office (PPL 70/8883). Three ChAT-IRES-Cre (JAX006410) mice were used (female, 8–37 weeks-old) and housed individually in high-roofed cages with a 12 h:12 h light/dark cycle (light on hours: 7:00–19:00). Mice had *ad libitum* access to food and water. All experiments were performed during the light period. No blind and randomized experimental design was adopted due to the nature of the technical development study.

### Surgery

The surgical procedures have been described previously (Tsunematsu et al., 2020). Briefly, mice were anesthetized with isoflurane (5% for induction, 1–2% for maintenance) and placed in a stereotaxic apparatus (SR-5M-HT, Narishige). Body temperature was maintained at 37°C with a feedback temperature controller (40–90–8C, FHC). Lidocaine (2%, 0.1–0.3 mg) was administered subcutaneously at the site of incision. Two bone screws were implanted on the skull for monitoring cortical EEGs and twisted wires were inserted into the neck muscle for obtaining EMG signals. An additional bone screw was implanted over the cerebellum to provide a ground/reference channel. These electrodes were connected to a two-by-three piece connector (SLD-112-T-12, Samtec). Two additional anchor screws were implanted bilaterally over the parietal bone to provide stability and a small portion (approximately 1 cm long) of a drinking straw was placed horizontally (opening facing medial/lateral axis) between the anchor screws and the connector. The purpose of the drinking straw was to create a hollow cavity within the head cap which allowed an Allen key to pass through and hold the mouse head still for connecting and disconnecting the mouse to the head-amp. The Allen key was securely clamped in place with a workbench vice. The viral vector (AAV5-CAG-flex-GCaMP6s-WPRE-SV40, Penn Vector Core; titer 8.3 × 10^12^ GC/ml) was microinjected (500 nl at 30 ml/min) (Nanoliter2010, WPI) to target the pedunculopontine tegmental nucleus (PPT) and laterodorsal tegmental nucleus (LDT) (−4.5 mm posterior, 1 mm lateral from bregma, and 3.25 mm depth from brain surface). The micropipette was left in the brain for an additional 10 min and then slowly raised up. A hybrid implant (see above) was then implanted 3 mm deep from the surface of the brain and all components were secured to each other and the skull with dental cement.

### Recording Procedures

After a recovery period (3–4 weeks), mice were habituated to being handled and tethered to the freely behaving system over several consecutive days. Mice were scuffed and the straw on the head cap slotted into a custom-made clamp, to keep the head still and absorb any vertical forces when connecting the electrophysiology and fiber photometry tethers to the head cap. Once connected, mice were placed in an open top Perspex box (21.5 cm × 47 cm × 20 cm depth) lined with absorbent paper, bedding, and soft food (creamed porridge, Heinz). During the habituation period, short recordings (20–30 min) were taken to test illumination parameters for the best signal to noise ratio. The illumination power was adjusted at the tip of optical fiber to 0.4–0.94 mW/mm^2^ for the 405 nm LED and 0.7–1.37 mW/mm^2^ for the 470 nm LED. Following the habituation period, simultaneous electrophysiological recording and calcium imaging was performed for 4–5 h to allow for multiple sleep/wake transitions.

### Histology

After electrophysiological experiments, animals were deeply anesthetized with mixture of pentobarbital and lidocaine and perfused transcardially with 20 ml saline followed by 20 ml 4% paraformaldehyde/0.1 M phosphate buffer, pH 7.4. The brains were removed and immersed in the above fixative solution overnight at 4°C and then immersed in a 30% sucrose in phosphate buffer saline (PBS) for at least 2 days. The brains were quickly frozen and were cut into coronal sections with a sliding microtome (SM2010R, Leica) with a thickness of 50 or 80 μm. To verify GCaMP6s expression in cholinergic neurons within the brainstem, sections were stained for choline acetyltransferase (ChAT) and green fluorescent protein (GFP). Brain sections were first washed (5 min, three times) at room temperature (RT) with PBS-Triton-X (PBST, 0.1 M PBS and 0.3% Triton-X) and then incubated in a blocking solution (10% normal donkey serum, NDS, Sigma-Aldrich, D9663 in 0.3% PBST) for an hour at RT. Next, sections were incubated with primary antibodies against GFP (mouse anti-GFP 1:2000, Abcam, ab1218) and ChAT (goat anti-ChAT 1:400, Millipore, AB144P) in PBST and 3% NDS overnight at 4°C. Sections were washed with PBS (5 min, three times) and incubated in a secondary antibody solution (donkey anti-mouse IgG alexa fluor 488, 1:500, Thermo Fisher Scientific, SA5-10166; Donkey anti-goat IgG alexa fluor 568, 1:500, Invitrogen, A11057) in PBST and 3% NDS for 2 h at RT. Sections were washed with PBS (20 min, two times) and then incubated with a 1/5000 solution of DAPI (Thermo Fisher Scientific) in PBS for 5 min. After washing (30 min, PBS at RT), sections were mounted on glass slides with gelatine solution. Slides were left to air dry and cover slipped. Sections were observed with an epifluorescence microscope (Nikon Eclipse E600, Grayscale).

### Signal Processing

All signal processing was performed offline using MATLAB (version 2018b, MathWorks).

#### Fiber Photometry

**FIGURE 3:**
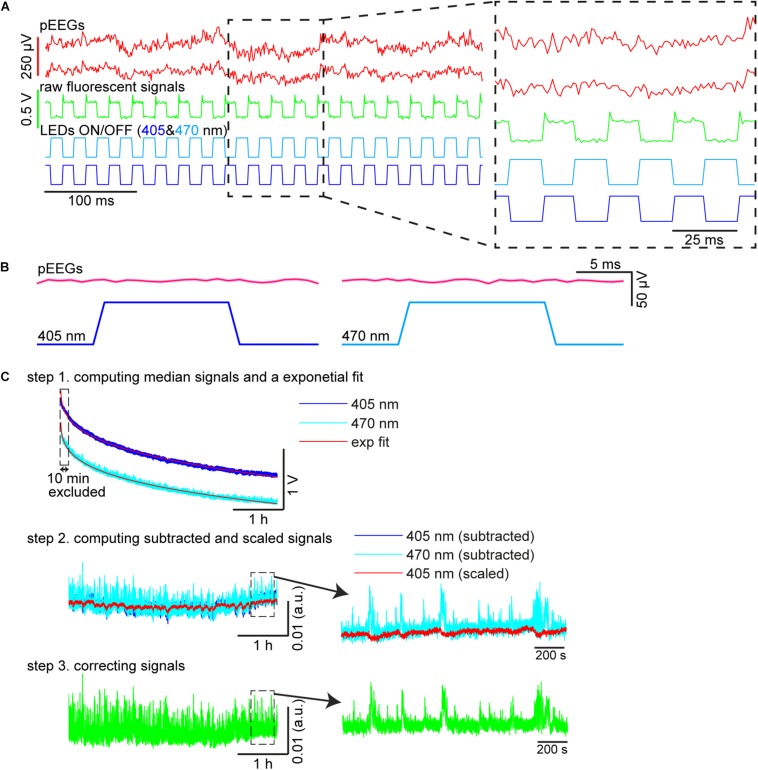
Signal processing. **(A)** Raw pontine EEGs (pEEGs) traces, raw fluorescent signals, and LED illumination patterns. **(B)** Averaged pEEG signals triggered by 405 nm (*left*) and 470 nm (*right*) excitation pulses in a 20-s recording (∼800 pulses). No noticeable artifact was observed. **(C)** Signal processing. (*top*) Medians were computed from raw fluorescent signals during individual illumination periods for each LED. The profile of the median values shown is over a 4-h recording period. *blue*, signals with 405 nm illumination. *light blue*, 470 nm illumination. *red*, exponential fit to estimate photobleaching. (*middle*) The median values in the top panel were subtracted from their exponential curves fitted, respectively (*blue*, 405 nm; *light blue*, 470 nm). The subtracted 405 nm signals were then linearly scaled (*red*) to evaluate moving artifacts. (*bottom*) The subtracted 470 nm signals in the middle panel were corrected by subtracting signals from the scaled 405 nm signals to provide normalized fluorescent signals.

Custom-written MATLAB scripts were used to compute fluorescent signals (Figure 3C). To extract 405 and 470 nm signals, illumination periods were determined by detecting synchronization ON/OFF pulses for each LED (see also Figure 3A). The median fluorescent signal was calculated during each illumination epoch (Figure 3C, step 1). Because each illumination epoch consisted of pulses at 40 Hz, the fluorescent signals originally sampled at 1 kHz were effectively down-sampled to 40 Hz. Photobleaching was estimated by fitting a single exponential curve and the difference between the fluorescent signal trace and the estimate was further low-pass filtered at 4 Hz given the slow kinetics of GCaMP6s (Figure 3C, step 2). To estimate moving artifacts, the filtered 405 nm signals were normalized based on the filtered 470 nm signals using a linear regression (Figure 3C, step 2). To estimate fluorescent signals, the fitted 470 nm signals were subtracted from the scaled 405 nm signals (Figure 3C, step 3).

#### Electrophysiology

Vigilance states were visually scored offline as described elsewhere (Tsunematsu et al., 2020). Wakefulness, NREM sleep, or REM sleep was determined over a 4-s resolution, based on cortical EEG and EMG signals using a custom-made MATLAB Graphical User Interface. The same individual scored all recordings for consistency.

**FIGURE 4:**
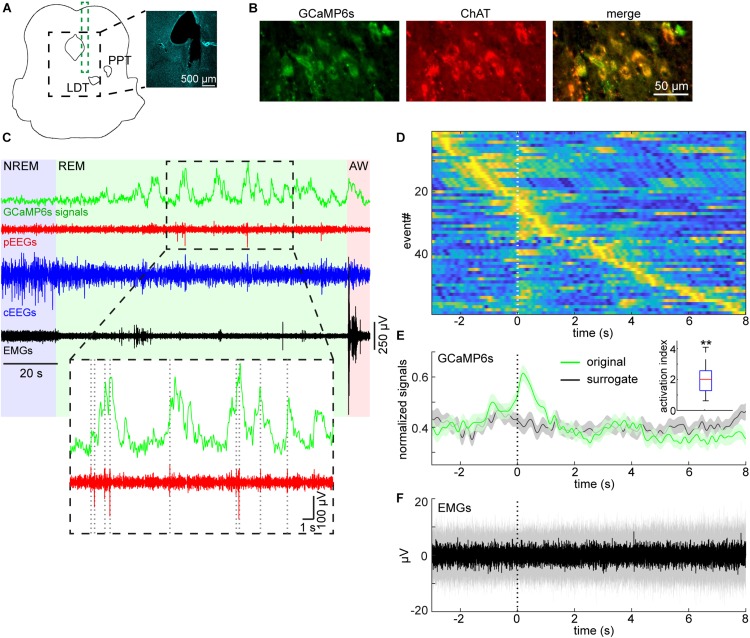
Pontine waves and mesopontine cholinergic activity during REM sleep. **(A)** Histological analysis of the position of an implanted optrode. The photograph is a DAPI stained image. LDT, laterodorsal tegmental nucleus. PPT, pedunculopontine tegmental nucleus. **(B)** Expression patterns of GCaMP6s and ChAT. **(C)** Example GCaMP6s, pontine EEGs (pEEGs), cortical EEGs (cEEGs), and EMGs signals around a REM sleep episode. Inset, pontine waves (spiking events in pEEGs) often co-occurred with large activity of calcium signals. **(D)** GCaMP6s signals triggered by detected P-waves. Time zero indicates the onset of pontine waves (P-waves) during REM sleep. GCaMP6s signals were normalized by the maximum value of each trace and all traces were sorted by the peak timing of GCaMP6s signals. **(E)** The mean profile of calcium signals triggered by detected P-waves. The normalized signals in **(D)** were averaged (green). In the surrogate condition (black), the timing of P-waves were randomly assigned during each REM sleep episode whilst still conserving the number of P-waves during the episode. Then the averaged calcium signals were computed. Errors, SEM. *Inset*, activation index in 1-s window from the onset of P-waves. **p < 0.01 (*t*-test). **(F)** The median profile of EMG signals triggered by detected P-waves. Shaded area, 25–75 percentile.

To detect P-waves, the two EEG signals from the pons were subtracted and filtered (5–30 Hz band-pass filter). If the signals crossed a threshold, the event was recognized as P-waves. To determine the detection threshold, a 1-min segment of the subtracted EEG signals was extracted from the longest NREM sleep episode to estimate stable noise level. The noise level was estimated by computing root-mean-square (RMS) values in every 10 ms time window. The threshold was defined as mean + 5 × the standard deviation of the RMS values. The timing of P-waves was defined as the timing of the negative peak. To generate surrogate P-wave timing during REM sleep (Figure 4E), the number of P-waves during each REM sleep episode was held, but P-wave timing was randomly allocated during the episode. This surrogate timing was used to extract GCaMP6s signals for comparisons. To assess the reproducibility of our observation in Figure 4, the activation index was defined as $\frac{F_{\text{real}}-F_{\text{surrogate}}}{\left|F_{\text{surrogate}}\right|}$, where *F*_*real*_ and *F*_*surrogate*_ were average real and surrogate fluorescent signals in 1-s window from the onset of P-wave, respectively.

### Statistical Analysis

Data was presented as mean ± SEM unless otherwise stated. Student’s *t*-test was performed in Figure 4E (*inset*).

## Results

### Simultaneous Monitoring of Pontine EEGs and Calcium Transients in Cholinergic Neurons in Freely Behaving Mice

First, we evaluated whether our system is suitable for a long-term recording over several hours from mesopontine cholinergic neurons along with electrophysiological recording. To this end, we collected six datasets from three animals. The average recording duration was 253.5 ± 8.5 min (range, 212.7–267.1 min) (Table 2). Figure 3A shows representative raw traces of pontine EEGs, raw fluorescent signals, and LED illumination pulses. Our initial concern was that optical illumination might induce optical artifacts in pontine EEG signals as reported in optogenetic experiments (Kozai and Vazquez, 2015). However, no optical artifact was observed (Figure 3B).

**TABLE 2:** Statistics of sleep-wake cycles in individual recordings.

Animal #	Rec #	Duration (min)				# of episodes			Episode duration (min)		
				
		Total	NREM	REM	AW	NREM	REM	AW	NREM	REM	AW
1	1	266.9	115.2	8.2	143.5	110	6	110	1.0 ± 0.9	1.4 ± 1.0	1.3 ± 1.9
1	2	265.0	100.7	1.7	162.6	107	2	107	0.9 ± 0.7	0.8 ± 0.4	1.5 ± 2.4
1	3	256.9	103.6	4.9	148.3	134	7	134	0.8 ± 0.6	0.7 ± 0.5	1.1 ± 1.5
1	4	212.7	100.2	8.9	103.6	108	6	108	0.9 ± 0.9	1.5 ± 1.2	0.9 ± 1.2
2	1	267.1	98.9	1.6	166.6	143	2	143	0.7 ± 0.5	0.8 ± 0.8	1.2 ± 2.7
3	1	252.7	47.2	1	204.5	76	2	76	0.6 ± 0.5	0.5 ± 0.0	2.6 ± 5.7

*AW, wakefulness; REM, rapid eye movement sleep; NREM, non-REM sleep. Data represents mean ± standard deviation.*

We also evaluated the stability of calcium transient amplitudes during the recording. While overall fluorescent signals decreased exponentially (Figure 3C), calcium transients were robust over several hours (Figure 3C). Thus, our approach allows for the simultaneous monitoring of both electrophysiological signals and calcium transients in freely behaving animals across the sleep-wake cycle.

### Pontine Waves and Calcium Transients in Mesopontine Cholinergic Neurons

We recently reported P-waves in mice (Tsunematsu et al., 2020). Because mesopontine cholinergic neurons have been implicated in P-wave genesis (Callaway et al., 1987; Datta, 1997), we examined whether P-waves co-appear with calcium transients in mesopontine cholinergic neurons during REM sleep. Figures 4A,B represent the position of the optrode and co-expression of GCaMP6s and ChAT, respectively. Example signals around a REM sleep episode is shown in Figure 4C. We observed frequent calcium transients during REM sleep as well as phasic, large fluctuations of pEEGs. These signals were qualitatively similar to those which were separately observed in our previous study (Tsunematsu et al., 2020).

We then quantified whether these two events co-occur across recordings (Figures 4D,E). We found that large calcium transients appeared around the timing of P-waves (Figure 4D). To quantify this trend, we computed the average fluorescent signals and compared this with surrogate signals across all detected P-wave events (Figure 4E). The calcium transient from the real data was larger than that from the surrogate data and this trend was consistent across all six recordings (Figure 4E, *inset*) (*p* < 0.01, *t*-test). In addition, we did not observe changes in EMGs associated with P-waves (Figure 4F), indicating that these transients and P-waves were not due to movement artifacts. Thus, we confirmed that P-waves co-occurs with calcium transients in mesopontine cholinergic neurons during REM sleep.

## Discussion

A combination of electrophysiological recording and calcium imaging can be used to monitor cell-type-specific activity together with sub-second neuronal events in freely behaving animals. In this study, we utilized this approach to correlate calcium transients in mesopontine cholinergic neurons with P-waves during REM sleep in mice for the first time. The same approach can be applied in various experimental contexts. Thus, our method adds a novel tool to investigate state-dependent neural circuit dynamics *in vivo*.

### Comparisons With Existing Systems

Although there are a handful of commercially available fiber photometry systems, our system is easy-to-build and economical. All parts of our fiber photometry system can be purchased from well-known suppliers and cost approximately 6,800 USD in total. Our *in vivo* electrophysiological recording system can be built with an additional budget of up to 6,000 USD. Therefore, our system offers an affordable solution to integrate *in vivo* electrophysiology with calcium imaging in freely behaving rodents. One of the main considerations to be made when setting up a fiber photometry system is whether a lock-in amplifier is required. Due to our offline analysis pipeline (Figure 3C), our system does not require a lock-in amplifier and thus drastically reduces costs as they typically costs over 5,000 USD. Although direct comparisons with commercially available systems are not straightforward due to differences in their specifications, several commercial systems (e.g., Doric Lens) offer a photometry system virtually equivalent to our system without the added functionality of electrophysiological recording and cost around 10,000 USD. Others with lock-in amplification are more expensive, but some of the more sophisticated commercial systems (e.g., RZ10x, Tucker-Davis Technologies) offer a multi-color, multi-channel options, which may be attractive to some of users.

A limitation of fiber photometry in general is that it provides only population-level activity. Although an alternative approach is the use of GRIN lenses (Ghosh et al., 2011; Skocek et al., 2018; Aharoni and Hoogland, 2019), this approach is more invasive due to larger lens diameters. Hence, simultaneous calcium imaging of individual neurons and electrophysiological monitoring may be challenging.

### Implications of Findings

In our recent study (Tsunematsu et al., 2020), we performed *in vivo* electrophysiological recordings of P-waves and GCaMP6s-based fiber photometry in mesopontine cholinergic neurons, separately. In the present study, we investigated the temporal relationship between population activity in mesopontine cholinergic neurons and P-waves during REM sleep by simultaneously monitoring both signals. Although P-waves have been studied in several mammalian species, such as cats, monkey, and rats since the 1960s, few studies have investigated P-waves in mice (Tsunematsu et al., 2020). Previous studies suggest that cholinergic neurons play a role in the induction of P-waves (Callaway et al., 1987; Steriade et al., 1990; Datta et al., 1992; Datta, 1997). In line with this, our results directly demonstrated that indeed mesopontine cholinergic population activity co-occurs with P-waves for the first time. A limitation of GCaMP6s is that it provides only an approximate reflection of neuronal spiking activity. Therefore, the exact temporal relationship between the firing of cholinergic neurons and P-waves still need to be investigated with the use of genetically encoded voltage indicators or electrophysiological techniques with optogenetic tagging. In addition to cholinergic neurons, it would be also interesting to monitor calcium transients in different cell types across pontine nuclei to characterize neural ensemble dynamics underlying P-waves.

### Future Directions

A similar approach can be taken in different experimental settings. For example, field potentials can be monitored with cell type-specific calcium transients in task performing animals. Our system can be customized to add optogenetic stimulation by expressing red-shifted indicators and opsins sensitive to blue light (Chen et al., 2013). A bipolar electrode can be replaced by other types of electrodes to record broadband signals including spiking activity to correlate calcium transients with neuronal spiking because various optrodes have been developed for optogenetic experiments (Ono et al., 2018; Sileo et al., 2018; Wang et al., 2018). The fiber photometry system can be updated to utilize a tapered optic fiber to monitor activity from a larger area (Pisanello et al., 2019) or to perform cell-type-specific voltage imaging (Marshall et al., 2016; Kannan et al., 2018). In conclusion, our combinatory approach with electrophysiological recording and fiber photometry offers an affordable, but powerful solution to interrogate state-dependent neural circuit dynamics across various brain regions and behavioral states.

## Data Availability Statement

The data for this article can be found at https://doi.org/10.15129/c7bb43e9-ffa5-490b-9fb2-41250c2ce449.

## Ethics Statement

The animal study was reviewed and approved by the United Kingdom Home Office (PPL 70/8883) and all protocols were performed in accordance with the Animals (Scientific Procedures) Act of 1986.

## Author Contributions

AP and SS designed and conceived the project and analyzed the data. AP and NM developed the recording system. AP performed all experiments. NM created the construction manual of the photometry system. AP, NM, and SS wrote the manuscript. KM and SS supervised NM and AP, respectively.

## Conflict of Interest

The authors declare that the research was conducted in the absence of any commercial or financial relationships that could be construed as a potential conflict of interest.

## Footnotes

1 https://github.com/Sakata-Lab
