# Supplementary material for: Simultaneous Electrophysiology and Fiber Photometry in Freely Behaving Mice
Source: Front Neurosci. 2020 Feb 21;14:148. doi: 10.3389/fnins.2020.00148 (PMC7047771; doi:10.3389/fnins.2020.00148)
Supplement: Supplementary file 1 [file Data_Sheet_1.PDF]

Supplementary Materials:

Simultaneous electrophysiology and fiber photometry in freely behaving mice

Amisha A Patel[1], Niall McAlinden[2], Keith Mathieson[2], Shuzo Sakata[1]

[1] Strathclyde Institute of Pharmacy and Biomedical Sciences, University of Strathclyde, 161 Cathedral Street, Glasgow G4 0RE, UK

[2] Institute of Photonics, Department of Physics, SUPA, University of Strathclyde, Glasgow, G1 1RD, UK

Construction Guide: Fiber Photometry System

Contents

Materials 2

Step by step guide 3

Software 10

Materials

1. **Light sources**

Suitable LASER and LED sources are available. We chose to implement an LED based system due to the lower cost and availability of a wider range of wavelengths. A 405 nm source (M405L3, Thorlabs) was chosen to give a Ca^{2+} - independent isosbestic signal. A 470 nm source (M470L3, Thorlabs) was chosen to match the peak absorption of our GECI (GCaMP6). The illumination system was designed to allow for future upgrades including additional illumination channels and LEDs with different wavelengths to allow for voltage sensing. The LED light was collimated using an aspheric lens, f = 20 mm, NA = 0.54 (AL2520M-A, Thorlabs). A LED driver (LEDD1B, Thorlabs) was used to power the LEDs. The output power was modulated using an external signal from a DAQ device (NI USB-6211, National Instruments).

2. **Filtering**

To ensure that there was no cross talk between the excitation and emission bands light from the LEDs was filtered using bandpass filters (FB470-10 and FB405-10, Thorlabs). A longpass dichroic mirror/beamsplitter (DMLP425R, Thorlabs) was used to combine the 405 nm and 470 nm beams. To separate the excitation and emission path a filter designed for GFP was used (MD498 - GFP, Thorlabs). Finally an emission filter designed for GFP (MF525-39 - GFP, Thorlabs) was used to filter out any remaining excitation light before the detector. If additional fluorescent channels are needed the filtering system will require upgrading.

3. **Patch cable**

The light was coupled into a patch cable (M82L01, Thorlabs) using an aspheric lens, f = 20 mm, NA = 0.54 (AL2520M-A, Thorlabs). A fiber launch system was used to align the fiber optic (KT110/M, Thorlabs). Auto-fluorescence from within the patch cable is a key consideration with a fiber photometry system. The patch cable used initially had significant auto-fluorescence, however, this was bleached out by leaving the system on with the LEDs at full power for >24hrs.

4. **Detector**

Fluorescence light after emerging from the fiber was collimated by the same aspheric lens that coupled light into the fiber f = 20 mm, NA = 0.54 (AL2520M-A, Thorlabs). The light then passes through the filtering system before being focused by an aspheric lens f = 20 mm, NA = 0.54 (AL2520M-A, Thorlabs) onto the detector (NewFocus 2151, Newport). The detector signal was digitised using a DAQ device (NI USB-6211, National Instruments).

Step by step guide

1. Mount LEDs into cage mount (CP12, Thorlabs)

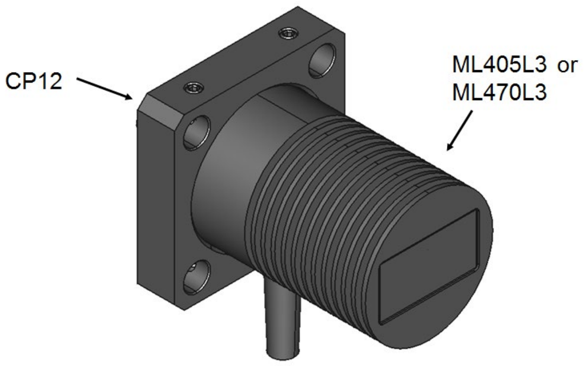

2. Mount aspheric lens into cage mounts (CP08/M, Thorlabs)
3. Fix the lens position relative to the LED to ensure the light emerging from the LED is collimated.
    a. Ensure that the flat surface of the lens is towards the LED.
    b. Positioning of the lens and further LED alignment can be completed with the LEDs at a low power to reduce the risk of eye damage.

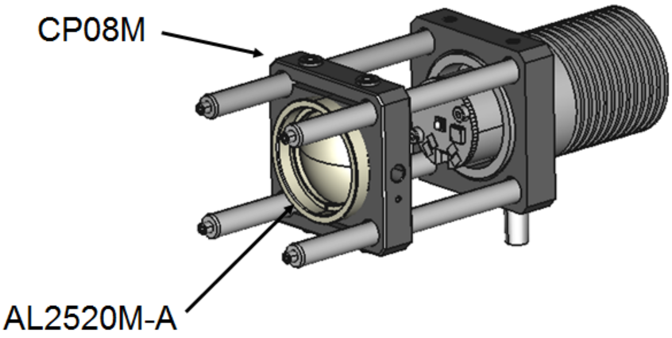

4. Mount the bandpass filters in the LED beam path

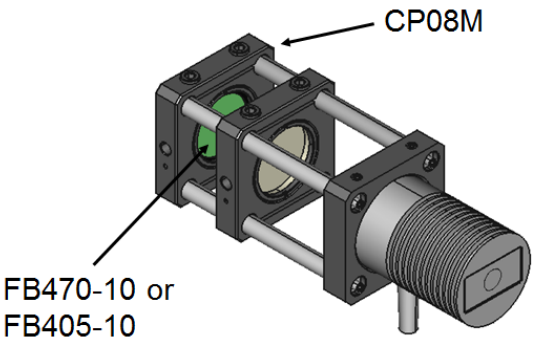

5. Mount the broadband mirrors (BB1-E02, Thorlabs) in the mirror mounts (KCB1C/M)
6. Mount the dichroic mirrors in the filter mounts (C4W, B4C/M and FFM1, Thorlabs)

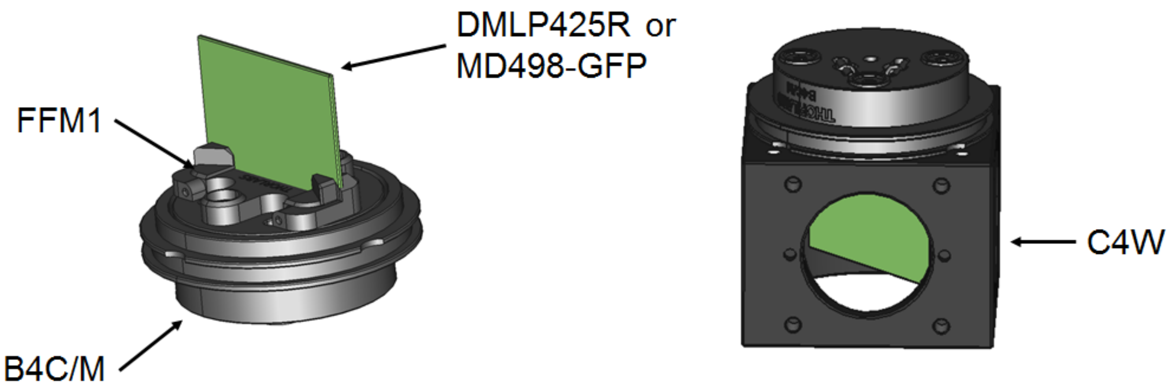

7. Fix the 470 nm LED to one of the mounts containing a broadband mirrors

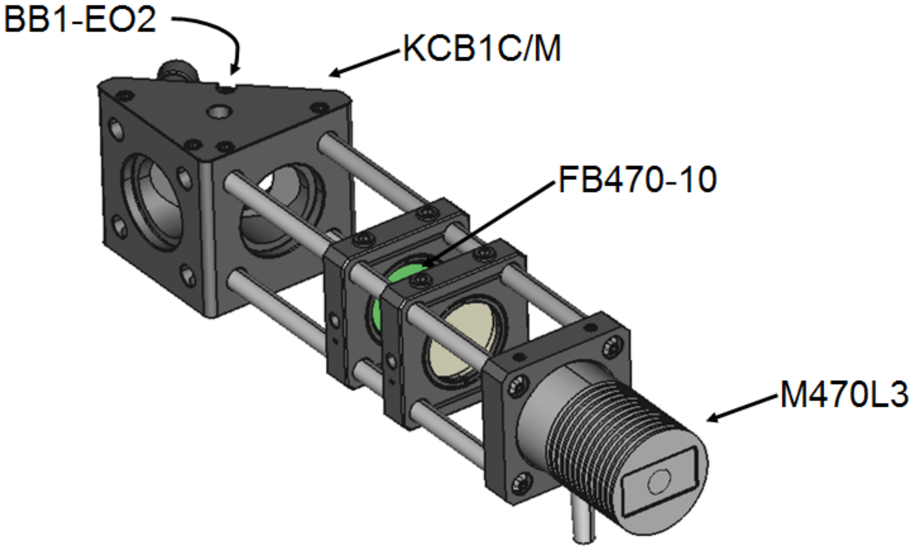

8. Fix the 405 nm LED to the dichroic mirror mount containing filter DMLP425R

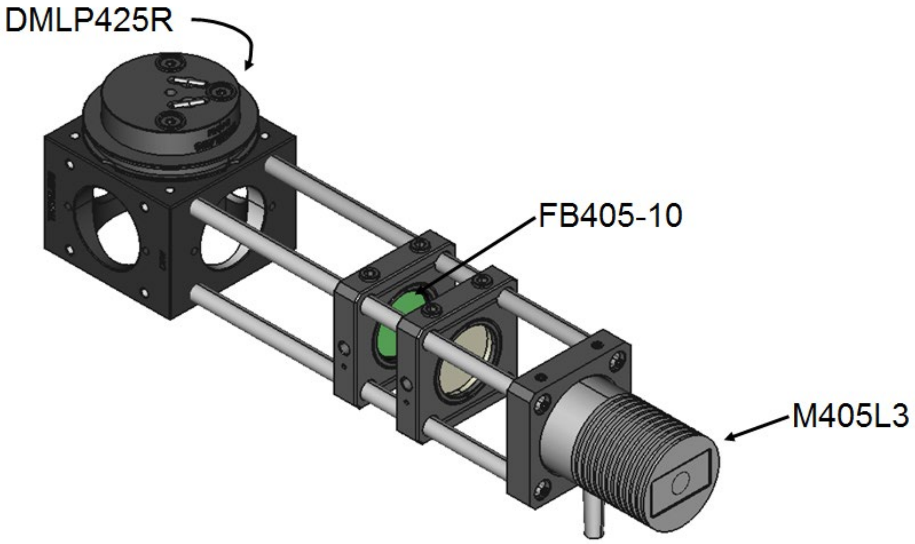

9. Fix the two mirror mounts together and co-align the beams using adjustment screws.

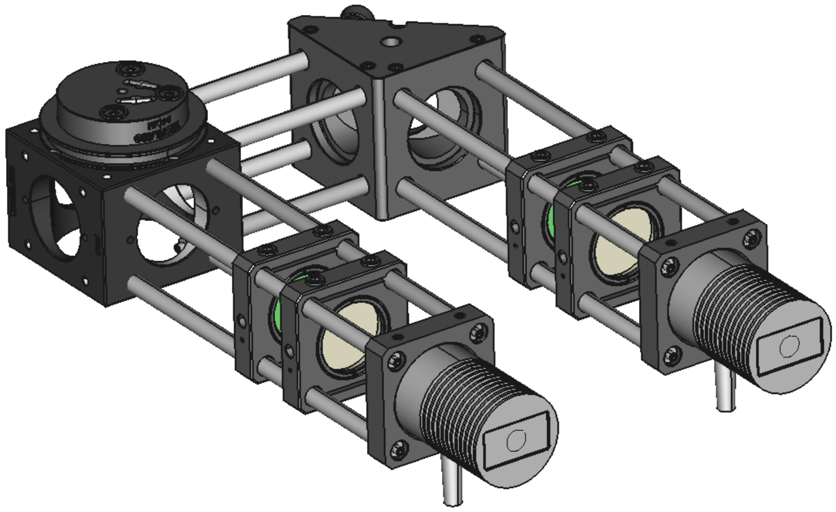

10. Fix the dichroic mirror mount containing filter MD498 - GFP to the system

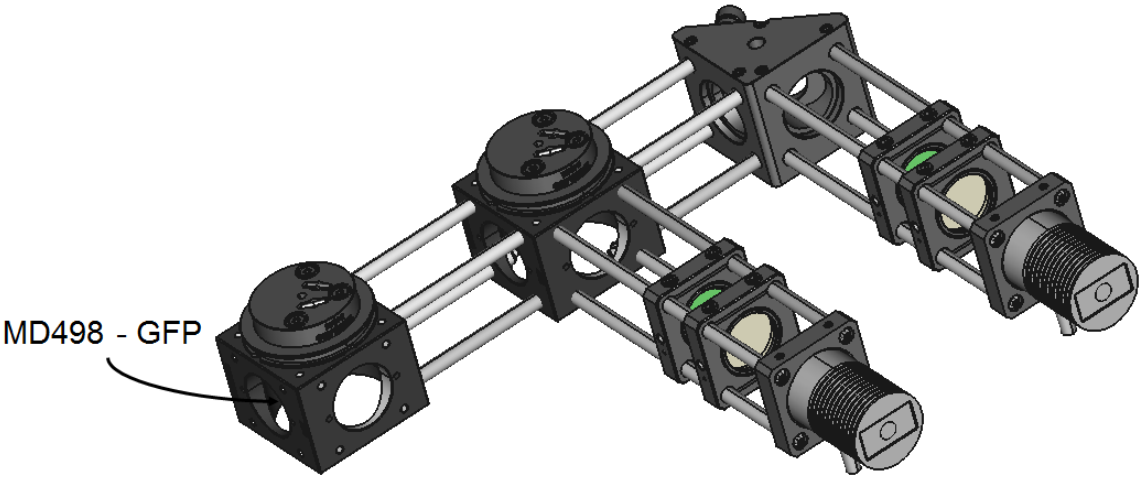

11. Fix an aspheric lens to the fiber launch system
    a. Ensure that the curved surface is towards the beam
12. Mount the fiber launch system

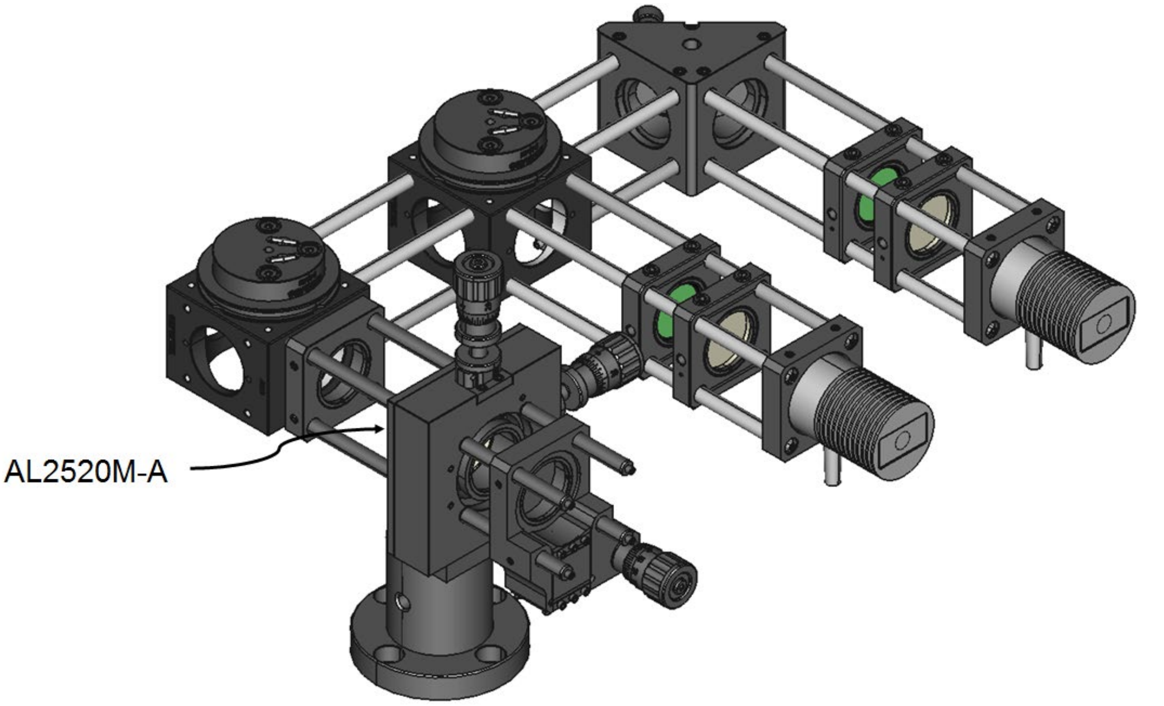

13. Align the focus of the beam to the core of the fiber optic.
    a. A photodetector can be used to measure the power out of the fiber optic and optimise the alignment

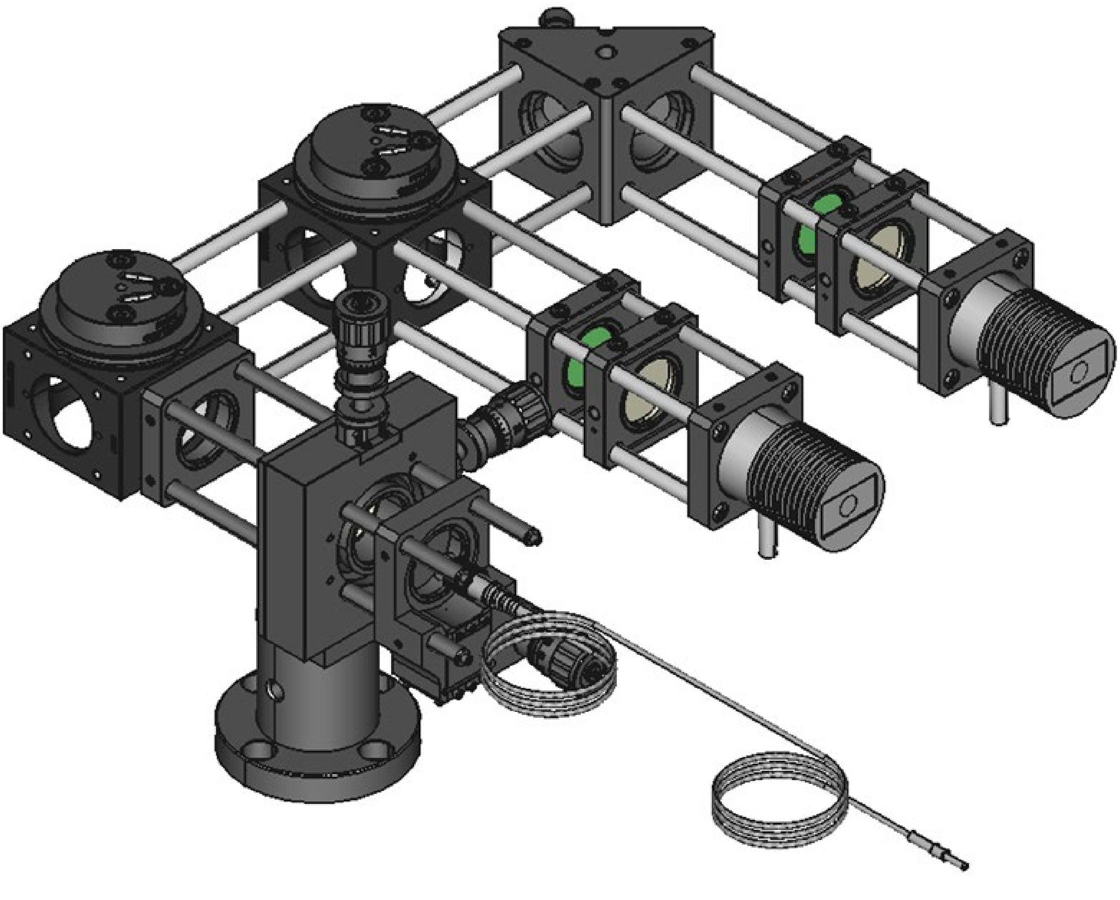

14. Place the far end of the fiber into fluorescein solution.
15. When the LEDs are turned up, it should be possible to see green light by eye after the dichroic (MD498 - GFP) mirror.

16. Mount the 2nd broadband mirror

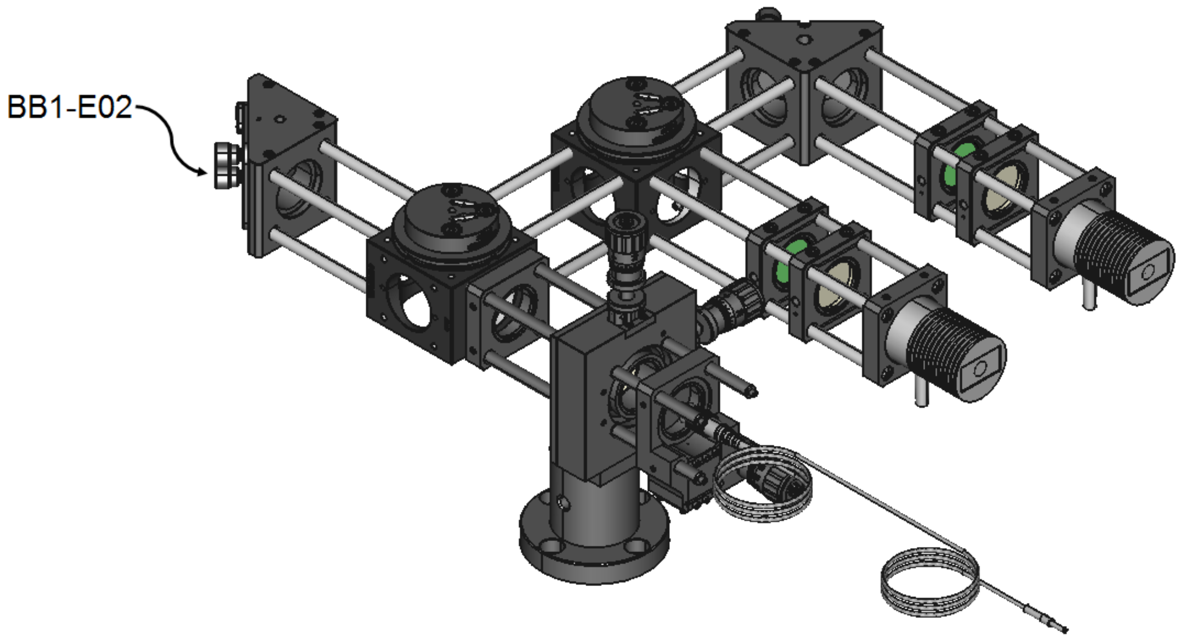

17. Mount the MF525-39 - GFP in the beam path

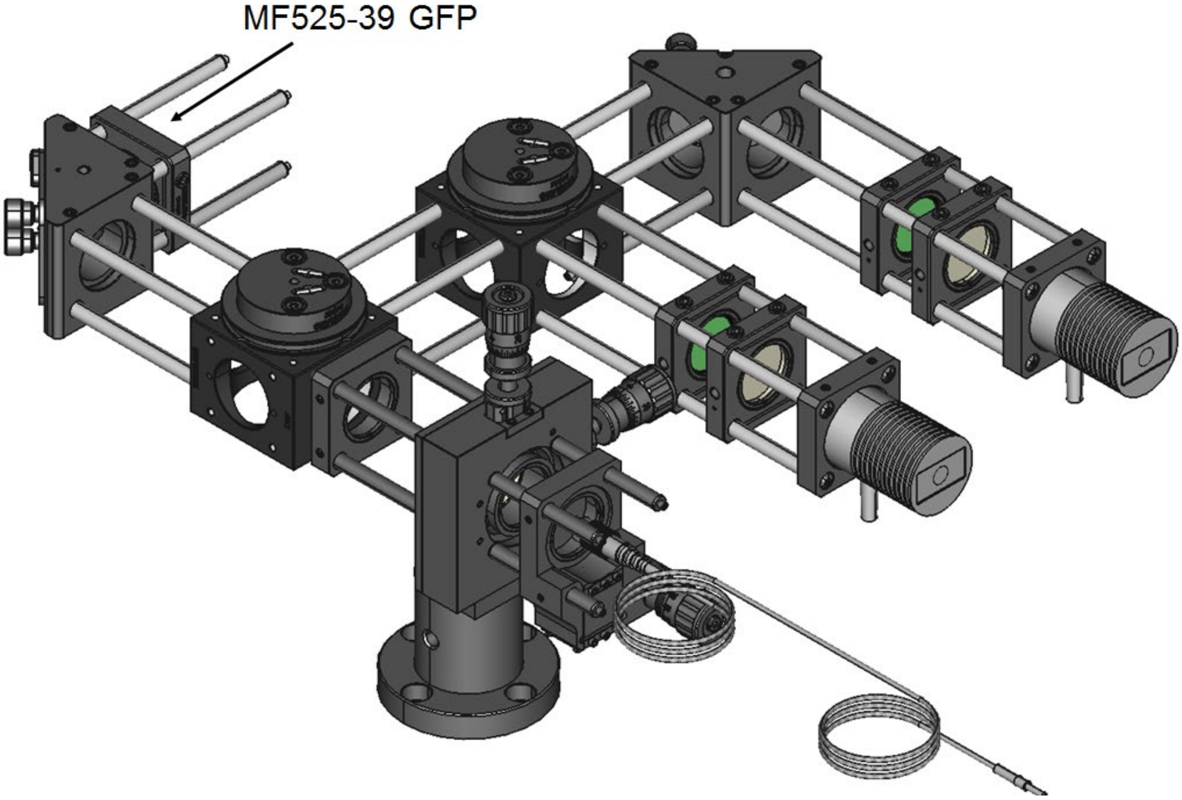

18. Mount the aspheric lens
    a. Ensure that the curved surface is towards the green beam

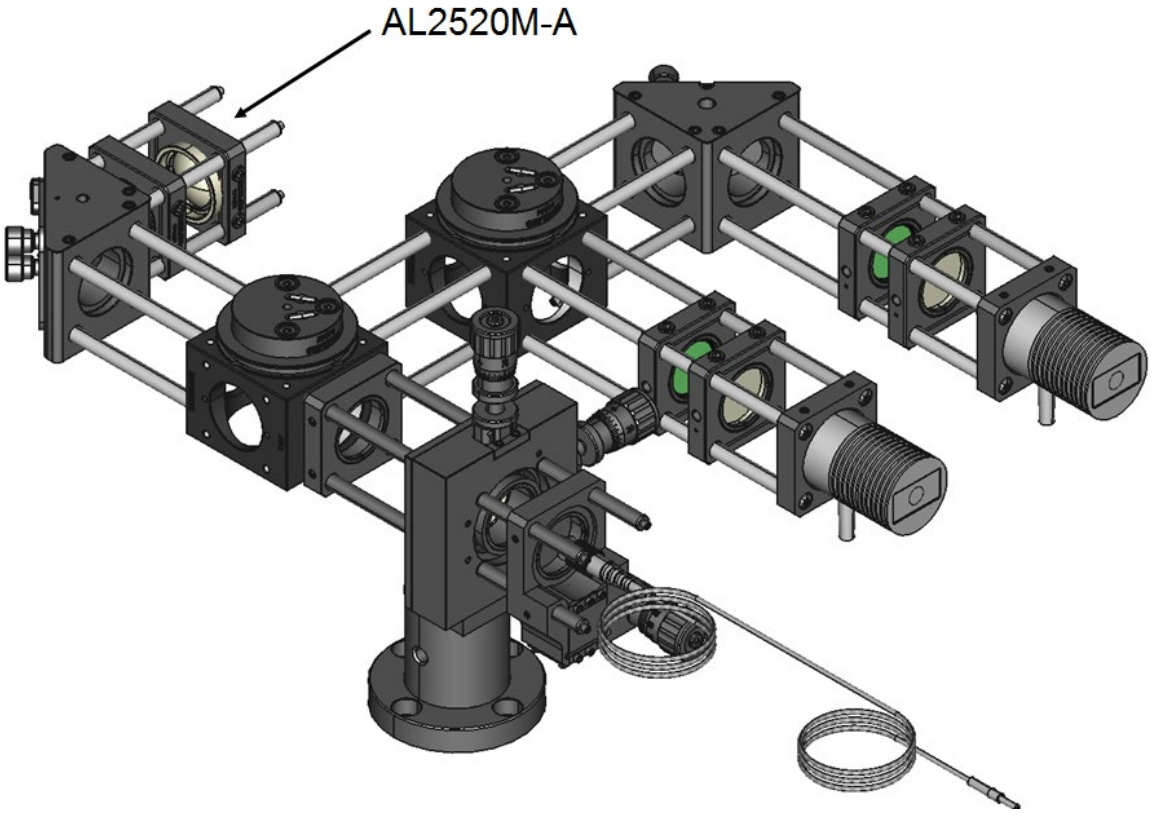

19. Mount the photodetector (NewFocus 2151, Newport).
    a. Align the photodetector and maximise the signal.
    b. It may be necessary to turn down the LEDs so that the detector does not saturate
    c. It may be necessary to work in a dark lab to prevent the detector saturating from background light.

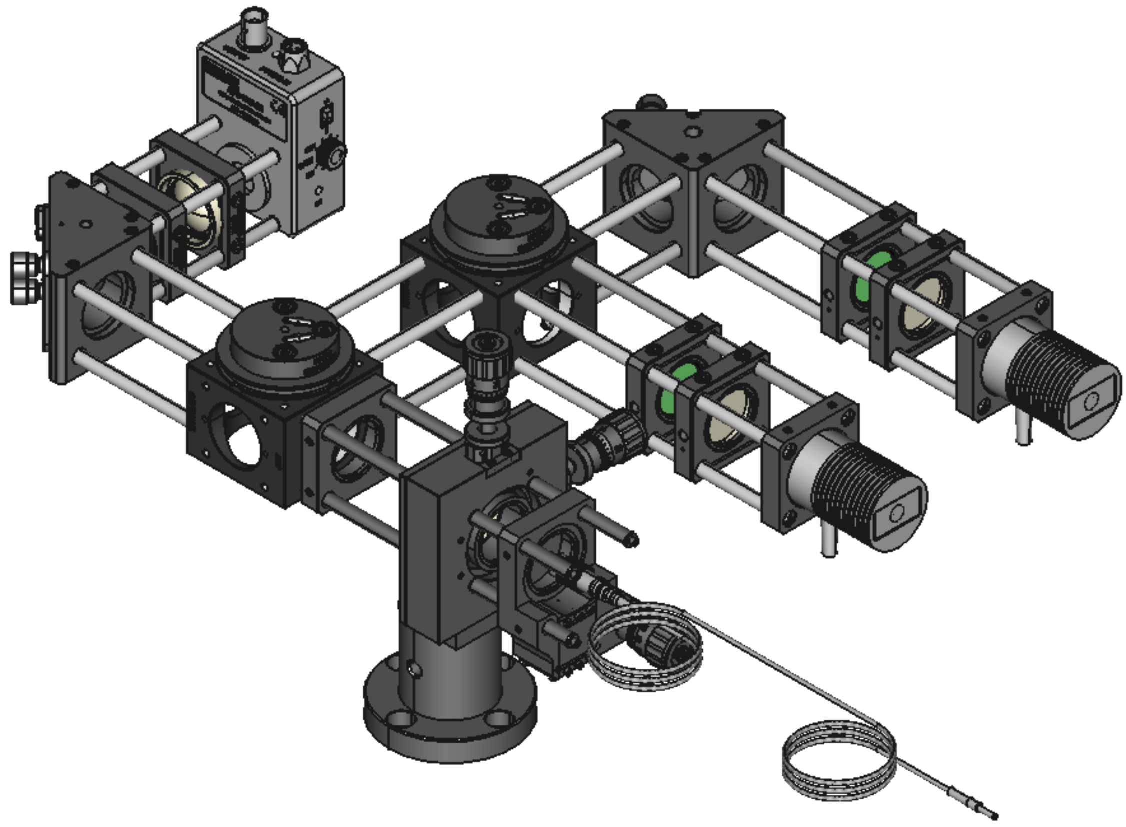

20. Mount lens tubes (SM1L03, Thorlabs) to shield background light from the emission pathway.

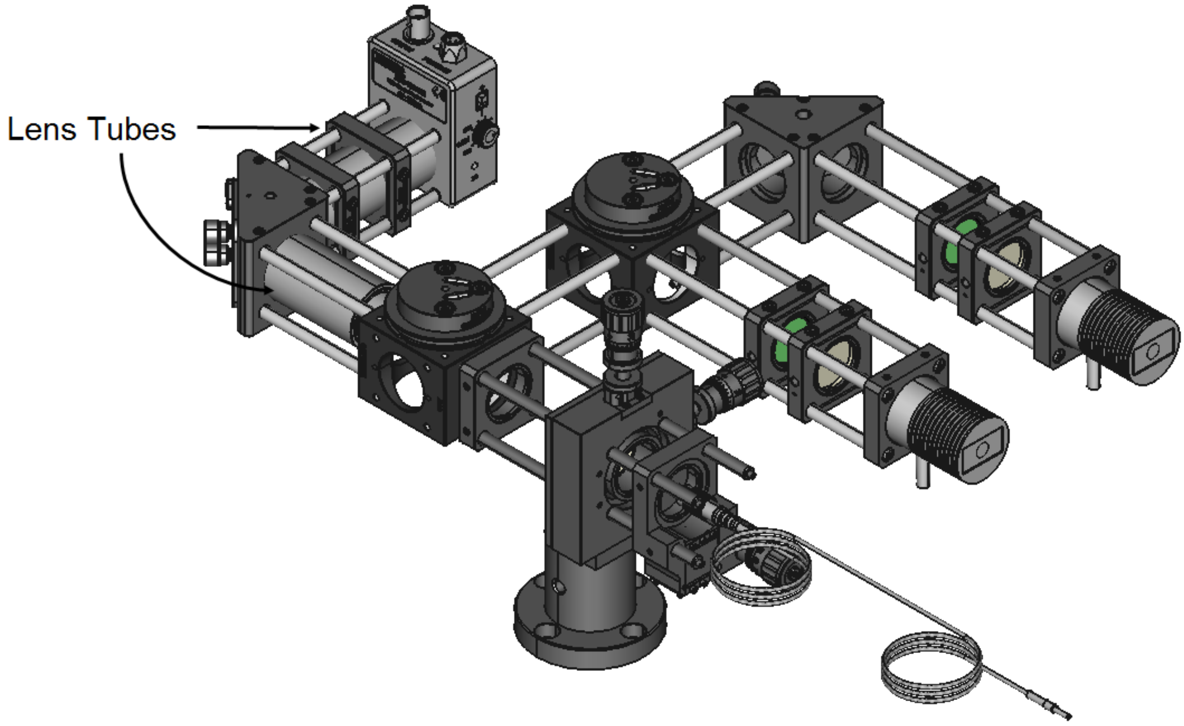

Software

Initially the intan system was started and recorded channels were routed to the analogue output on the RHD2000 device so that they could be recorded synchronously with the fiber photometry data.

The control software for the fiber photometry system was written in Labview 2018 (https://github.com/Sakata-Lab/ePhotometry). The software had 3 states; initialisation, data logging/visualization and shut down. The following processes took place in each states:

Initialisation:

1. Create file directory, data file, and file for experimental details
2. Set up LED waveforms.
    a. Each LED is pulsed with a square waveform at 40 Hz, the waveform has a 50% duty cycle. The two LEDs are 180º out of phase from each other.
    b. The LED current should be tuned so that each LED gives a similar fluorescent signal.
3. Initialise DAQ device
    a. Set up two analog outputs one for each LED
    b. Allow regeneration so that the LED waveform is continuous.
    c. Set up 3 analog inputs, one connected to the photodetector, with the other two connected to the analog outputs that are used to drive the LEDs
    d. Set up any additional analog inputs for recording the electrical data.

Data Logging/Visualization:

1. Record all analog input channels at 1000 Hz
2. Save the raw data to a binary file
3. Calculate the normalised fluorescence signal which is displayed on a graph.
    a. Record the signal on the detector when the 405 nm LED is on (S_{405}). This should be the average of 25 data points.
    b. Record the signal on the detector when the 470 nm LED is on (S_{470}). This should be the average of 25 data points.
    c. Take S_{470}/S_{405} to get the normalised fluorescence signal.
    d. Save the normalised fluorescence signal in a separate ANSI file so that it can be easily opened later
4. Display electrical recordings on an extra graph.

Shut down:

1. Stop and close all DAQ channels
2. Close all files

The front panel and block diagram of the Labview code are shown below.

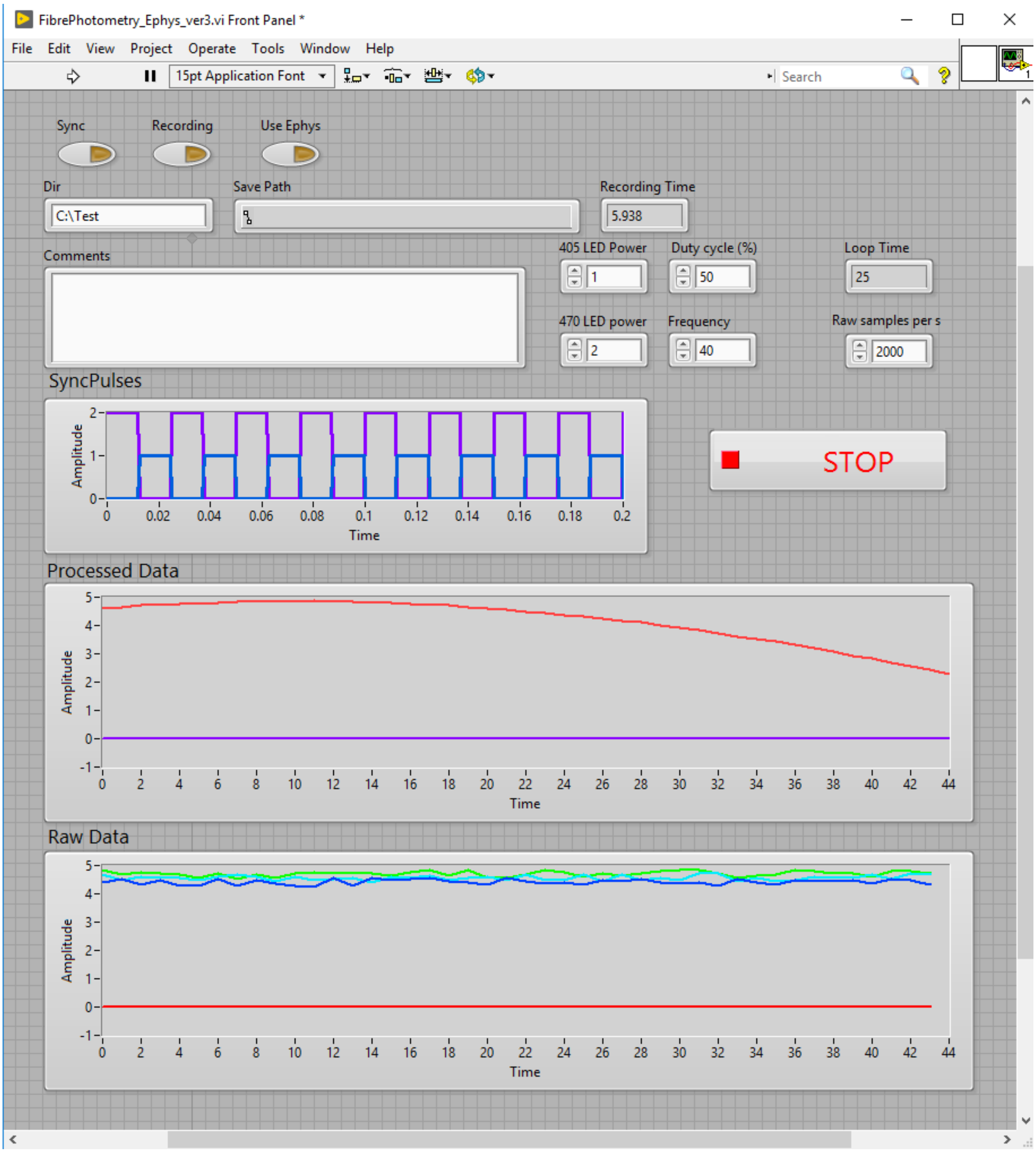
FibrePhotometry_Ephys_ver3.vi Front Panel *
File Edit View Project Operate Tools Window Help
15pt Application Font
Search
Sync
Recording
Use Ephys
Dir
C:\Test
Save Path
Recording Time
5.938
Comments
405 LED Power
1
Duty cycle (%)
50
Loop Time
25
470 LED power
2
Frequency
40
Raw samples per s
2000
SyncPulses
Amplitude
Time
STOP
Processed Data
Amplitude
Time
Raw Data
Amplitude
Time

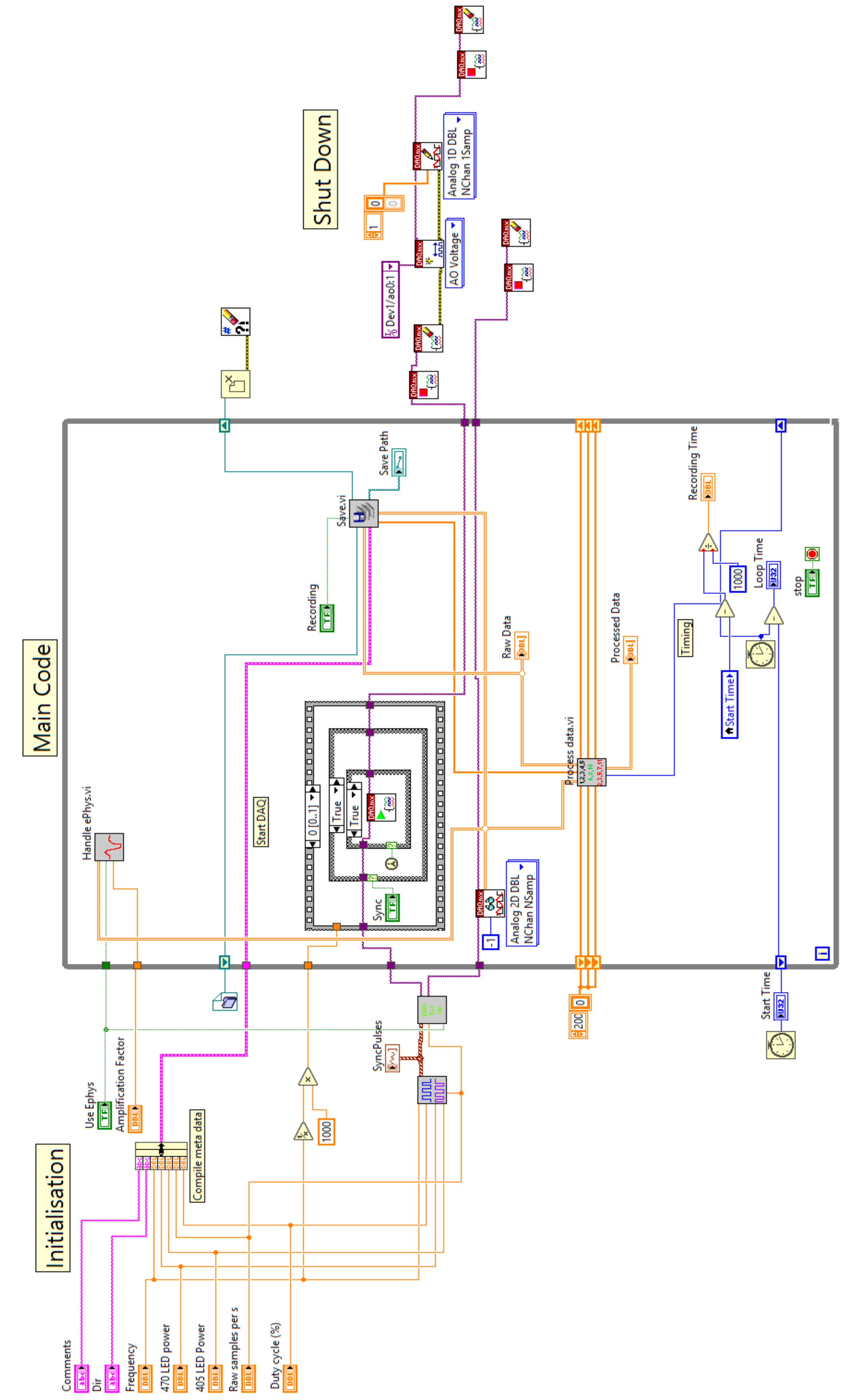
Initialisation
Main Code
Shut Down
Comments
Dir
Frequency
470 LED power
405 LED Power
Raw samples per s
Duty cycle (%)
Use Ephys
Amplification Factor
Compile meta data
1000
SyncPulses
Handle ePhys.vi
Start DAQ
Sync
Recording
Save.vi
Save Path
Raw Data
Processed Data
Process data.vi
Timing
Start Time
Loop Time
Recording Time
1000
stop
Analog 2D DBL
NChan NSamp
Analog 1D DBL
NChan 1Samp
AO Voltage
Dev1/ao0:1
True
200
0
